# TXNIP, a novel key factor to cause Schwann cell dysfunction in diabetic peripheral neuropathy, under the regulation of PI3K/Akt pathway inhibition-induced DNMT1 and DNMT3a overexpression

**DOI:** 10.1038/s41419-021-03930-2

**Published:** 2021-06-23

**Authors:** Xiang Zhang, Song Zhao, Qingqing Yuan, Lin Zhu, Fan Li, Hui Wang, Dezhi Kong, Jun Hao

**Affiliations:** 1 Department of Pathology, Hebei Medical University, Shijiazhuang, China; 2 Center of Metabolic Diseases and Cancer Research, Institute of Medical and Health Science of Hebei Medical University, Shijiazhuang, China; 3 Department of Electromyogram, The Third Hospital of Hebei Medical University, Shijiazhuang, China; 4 Department of Pharmacology of Chinese Materia Medica, Institution of Chinese Integrative Medicine, Hebei Medical University, Shijiazhuang, China

**Keywords:** Insulin signalling, Diabetes complications, Peripheral neuropathies

## Abstract

Diabetic peripheral neuropathy (DPN) is the most common complication of diabetes mellitus (DM) and the dysfunction of Schwann cells plays an important role in the pathogenesis of DPN. Thioredoxin-interacting protein (TXNIP) is known as an inhibitor of thioredoxin and associated with oxidative stress and inflammation. However, whether TXNIP is involved in dysfunction of Schwann cells of DPN and the exact mechanism is still not known. In this study, we first reported that TXNIP expression was significantly increased in the sciatic nerves of diabetic mice, accompanied by abnormal electrophysiological indexes and myelin sheath structure. Similarly, in vitro cultured Schwann cells TXNIP was evidently enhanced by high glucose stimulation. Again, the function experiment found that knockdown of TXNIP in high glucose-treated RSC96 cells led to a 4.12 times increase of LC3-II/LC3-I ratio and a 25.94% decrease of cleaved caspase 3/total caspase 3 ratio. Then, DNA methyltransferase (DNMT) inhibitor 5-Aza has been reported to benefit Schwann cell in DPN, and here 5-Aza treatment reduced TXNIP protein expression, improved autophagy and inhibited apoptosis in high glucose-treated RSC96 cells and the sciatic nerves of diabetic mice. Furthermore, DNMT1 and DNMT3a upregulation were found to be involved in TXNIP overexpression in high glucose-stimulated RSC96 cells. Silencing of DNMT1 and DNMT3a effectively reversed high glucose-enhanced TXNIP. Moreover, high glucose-inhibited PI3K/Akt pathway led to DNMT1, DNMT3a, and TXNIP upregulation in RSC96 cells. Knockdown of DNMT1 and DNMT3a prevented PI3K/Akt pathway inhibition-caused TXNIP upregulation in RSC96 cells. Finally, in vivo knockout of *TXNIP* improved nerve conduction function, increased autophagosome and LC3 expression, and decreased cleaved Caspase 3 and Bax expression in diabetic mice. Taken together, PI3K/Akt pathway inhibition mediated high glucose-induced DNMT1 and DNMT3a overexpression, leading to cell autophagy inhibition and apoptosis via TXNIP protein upregulation in Schwann cells of DPN.

## Introduction

Diabetic peripheral neuropathy (DPN) is the most common complication of diabetes mellitus (DM) and has a variety of clinical manifestations including neuropathic pain, autonomic nerves system dysfunction, sensory loss, and diabetic foot. At present, pharmacotherapy on DPN is limited to symptomatic treatment, and there is no effective causal treatment. Therefore, a better elucidation and understanding of the exact mechanism involved in the pathogenesis and development of DPN is necessary to find the promising approaches to treat this disease [1]. Schwann cell dysfunction was gradually realized in the pathogenesis of diabetic peripheral neuropathy. The accumulating data demonstrated that the metabolic disturbance of Schwann cells in diabetes mellitus (DM) caused neurotoxic intermediates accumulation and neurotrophic factors deficiency, in turn the damage of both the vasculature and axons, finally leading to DPN [2, 3]. However, the exact mechanism involved in Schwann cell dysfunction of DPN is still not known.

Thioredoxin-interacting protein (TXNIP) is known as an inhibitor of thioredoxin (TXN) to regulate the intracellular redox homeostasis. Also, reactive oxygen species (ROS) drives TXNIP to bind with NLRP3, leading to the inflammatory reaction [4]. Besides, TXNIP also exerts other redox-independent effects including aberrant glucose metabolism, senescence, apoptosis, immunity, and tumorigenesis [5]. TXNIP has been reported to be elevated in the multiple tissues in diabetes mellitus and has detrimental effects on cell function and metabolism [6]. Amin et al. found that the inhibition of ROS-TXNIP-NLRP3 inflammasome pathway with dimethyl fumarate (DMF) offered the vasculoprotective influences on diabetic aortas [7]. Lv et al. also revealed that sulforaphane (SF) can delay retinal photoreceptor cell degeneration in diabetes via inhibition of endoplasmic reticulum stress (ERS), inflammation, and TXNIP expression through AMPK pathway activation [8]. In diabetic nephropathy, TXNIP silencing attenuated high glucose-induced apoptosis of podocyte, which was related to the inhibition of p38 MAPK and mTOR signaling pathway [9]. TXNIP has been revealed to mediate neuropathic pain in partial sciatic nerve ligation (pSNL) mice under the control of miRNA-23a/CXCR4. Inhibition of TXNIP reversed neuropathic pain elicited by pSNL [10]. Also, serum TXNIP content was reported to be negatively associated with peripheral nerve conduction velocity (NCV) in patients with type 2 diabetes mellitus. Serum TXNIP was an independent risk factor for the decreased left ulnar motor conduction velocity (MCV), right ulnar MCV, left median MCV, and right median MCV [11]. Cheng et al. reported in vitro-cultured rat Schwann cells (RSC96) that loganin prevented cell pyroptosis by inhibiting TXNIP/NLRP3 inflammasome and ROS production [12]. Here, whether TXNIP mediated Schwann cell dysfunction of DPN is still not elucidated.

DNA methylation is a kind of epigenetic modification involved in multiple cell processes including aging, tumor progression, proliferation, apoptosis, autophagy, and immunity by regulating gene transcription and chromatin structure [13, 14]. The 5′ carbon of cytosine in sporadic CpG dinucleotide is common labeled with methyl mark, and that in CpG island is common non-methylated. The methylation of CpG islands located in the promoter of gene usually affects gene transcription [15]. DNA methyltransferases (DNMTs) including DNMT1, DNMT3a, and DNMT3b in mammals are responsible for the writing of methyl mark [16]. In DPN, DNMTs inhibitor 5-Aza had been proven to benefit Schwann cells and promoted brain derived neurotrophic factor (BDNF) expression [17].

PI3K/Akt pathway is one of the important intracellular signaling pathways to regulate the basic cell functions, such as cell proliferation, metabolism, autophagy, motility, and apoptosis. Akt is the main downstream effector of PI3K and activated by the phosphorylation at site Ser 473 and Thr 308 [18]. In DPN, PI3K/Akt pathway was reported to be obliterated [19] and hyperglycemia was the critical factor to lead to the inhibition of Akt phosphorylation in Schwann cells [20].

In this study, we revealed the expression of TXNIP was increased in the sciatic nerves of diabetic mice, and high glucose-stimulated rat Schwann cells (RSC96), human Schwann cells (HSC), and primary rat Schwann cells (PRSC). Then, it was showed that knockdown of TXNIP in high glucose-treated RSC96 cells and HSC cells avoided cell apoptosis and recovered cell autophagy. Again, application of DNA methyltransferases inhibitor 5-Aza inhibited TXNIP and Bax protein expression, and increased LC3-II/LC3-I ratio in RSC96 cells. Also, 5-Aza improved the sciatic nerves function in diabetic mice accompanied with apoptosis inhibition and autophagy recovery. Furthermore, DNMT1 and DNMT3a were found to be the main regulators in high glucose-cultured RSC96 cells as the targets of 5-Aza, affecting TXNIP expression. In addition, high glucose-inhibited PI3K/Akt pathway caused DNMT1, DNMT3a, and TXNIP upregulation in RSC96 cells. In vivo knockout of TXNIP in diabetic mice improved nerve conduction, myelin sheath structure and autophagy function of the sciatic nerves.

## Materials and methods

### Materials

Antibodies against TXNIP (ET1705-72), DNMT1 (ET1702-77) and DNMT3a (ET1609-31) were bought from HuaAn Biotechnology (Hangzhou, Zhejiang, China). Antibodies against S-100 (ab52642), LC3 (ab192890), MBP (ab7349) and Akt (ab8805) were purchased from Abcam Co., (Cambridge, MA, USA). Antibodies against Caspase 3 (19677-1-AP) and Bax (50599-2-Ig) were bought from Proteintech Group, Inc., (Chicago, IL). Antibodies against phospho-Akt (Ser 473) (#4060), phospho-Akt (Thr 308) (#13038) and cleaved Caspase 3 (#9661) were from Cell Signaling Technology Inc. (Boston, MA). The β‐actin antibody was from ABclonal Biotechnology Co., (Wuhan, Hubei, China). The 40, 6-Diamidino-2-phenylindole dihydro-chloride (DAPI, # D8417), streptozotocin (STZ, # S0130) and insulin (# I2643) were bought from Sigma Chemical Co., (St. Louis, MO). LY294002, 5-Aza, MG132 and chloroquine were bought from MCE Co. (Monmouth Junction, NJ, USA). Standard Process kit (SP9000) for immunohistochemistry was bought from Zhongshan Golden Bridge Biotechnology (Beijing, China). DyLight 405-labeled, 488-labeled, 594-labeled goat secondary antibodies were bought from KPL (Gaithersburg, MD, USA). PrimeScript^TM^ RT reagent Kit with gDNA Eraser and SYBR® Premix Ex Taq^TM^ II (Tli RNaseH Plus) were bought from Takara Co. (Otsu, Shiga, Japan). Lipofectamine 3000 transfection reagent was purchased from Thermo Fisher Scientific (Waltham, MA, USA). TXNIP expression plasmid (pcDNA3.1-TXNIP) was constructed from DNA fragments encoding TXNIP that was a gift from Professor Jiahuai Han (Xiamen University). The pcDNA3/Myc-DNMT1 and pcDNA3/Myc-DNMT3a were the gifts from Arthur Riggs (Addgene plasmid # 36939, # 35521). TXNIP shRNA plasmid (pGenesil-1-TXNIP), DNMT1 shRNA plasmid (pGenesil-1-DNMT1) and DNMT3a shRNA plasmid (pGenesil-1-DNMT3a) were constructed by our lab. The target sequences were respectively GCAGAAGATCAGACCGTCCAT (TXNIP), GTACTTACTCCAA GTTCAA (DNMT1) and GCCCAAGGTCAAGGAGATCA (DNMT3a).

### Animal studies

All animal experiments were approved by the Institutional Animal Care and Use Committee of Hebei Medical University. All mice were given free access to water and normal diet and housed with temperature (22–23 °C). PASS Sample Size Software was used to evaluate sample size. The grouping of experimental mice was performed by simple random sampling method and the relative detection was performed blindly. Male C57BL/6J mice, at 6–8 weeks of age were randomly divided into two groups: control mice and diabetic mice (six mice per group). Type I diabetic mice models were made by the intraperitoneal injection of streptozotocin (STZ, 150 mg/kg weight) in 50 mmol/L sodium citrate solution (pH 4.5). Those mice with the random blood glucose more than 16.7 mmol/L were considered as diabetic mice at 72 h after the induction. After 2 months, action amplitude and nerve conduction velocity (NCV) of sciatic nerves were measured in normal mice and diabetic mice to determine the presence of neuropathy, and then all mice were euthanized by exsanguination after deep isoflurane anesthesia for the further detections. To explore the in vivo effect of 5-Aza, diabetic mice were treated with 5-Aza at a 600 μg/kg weight by the intraperitoneal injection every other day, and the control mice were administrated with the corresponding solvent (male, six mice per group). Two months later, the relevant detections were carried out after all mice were sacrificed. Transcription activator-like effector nucleases (TALEN) method was used to generate TXNIP (−/−) mice in C57BL/6J background. Wildtype TXNIP (+/+) littermates were utilized as controls (male, six mice per group). At 2 months from STZ injection, the related detections were performed.

### Cell lines and groups

Rat Schwann cells (RSC96, ATCC^Ⓡ^ CRL-2765^TM^) and human Schwann cells (HSC, #BNCC338553, Beina Chuanglian Biotechnology, Beijing, China) were cultured in DMEM supplemented with 10% fetal bovine serum (FBS), 1% penicillin and 1% streptomycin. Primary rat Schwann cells (PRSC) were isolated from the sciatic nerves of neonatal rats (less than 4 days) and maintained under DMEM medium containing 10% FBS, 20 ng/mL BFGF, and 2 μmol/L forskolin. To explore the effect of high glucose, RSC96 cells, HSC cells, and PRSC cells were respectively divided into three groups: normal glucose group (5.5 mmol/L glucose), high glucose group (25 mmol/L glucose), and mannitol group (25 mmol/L mannitol). After cell cycle synchronization by serum deprivation, cells were respectively treated with normal glucose, high glucose, and mannitol for 48 h. To elucidate the function of TXNIP, cells were respectively transfected with pcDNA3.1, pcDNA3.1-TXNIP, control shRNA plasmid (pGenesil-1), and TXNIP shRNA plasmid (pGenesil-1-TXNIP). To explore the effect of 5-Aza, a 10 μmol/L 5-Aza was used for RSC96 cells. To investigate the effect of DNMT1 and DNMT3a, RSC96 cells were randomly divided into pcDNA3.1 group, pcDNA3.1-DNMT1 group, pcDNA3.1-DNMT3a group, pGenesil-1 group, pGenesil-1-DNMT1 group, and pGenesil-1-DNMT3a group. To elucidate TXNIP protein degradation, a 10 μmol/L MG132 and a 10 μmol/L chloroquine were used to treat RSC96 cells for 1 h. To figure out the effect of PI3K/Akt pathway, RSC96 cells were treated with a 20 μmol/L LY294002 or a 1 μg/mL insulin.

### Electrophysiology measurement

Electrophysiological recording was performed after mice were anesthetized as described previously [21]. Stimulating needle electrode and recording needle electrode were respectively placed on the proximal and distal ends of the sciatic nerves. The distance between these two electrodes was measured. Action amplitude and nerve conduction velocity (NCV) of the sciatic nerves were recorded to evaluate the strength and velocity of nerve conduction.

### Electron microscope

Myelin sheath structure and autophagosome of the sciatic nerves were detected by electron microscopy observation. In brief, the sciatic nerves were fixed in 2% glutaraldehyde in 0.1 M sodium phosphate buffer overnight at 4 °C. Then the tissues were post-fixed in 2% osmium tetroxide in 0.1 M phosphate buffer at 4 °C for 2 h. After dehydrated and embedded, the ultrathin sections were stained with 2% uranyl acetate and lead citrate, followed by the observation using an electron microscope.

### Immunohistochemistry

The expression of TXNIP, LC3, cleaved Caspase 3, and Bax in the sciatic nerves of mice was determined by immunohistochemistry (IHC) staining. After the slides were deparaffinized and hydrated, the slides were immersed in citric acid/sodium citrate solution for high pressure cooking antigen retrieval. Next, the slides were incubated with 0.3% H_2_O_2_ for 10 min to quench endogenous peroxidase, and treated by goat serum for 30 min to block the nonspecific binding. Subsequently, the slides were incubated with the primary antibody overnight at 4 °C. After incubated with biotin‐conjugated goat secondary antibody at 37 °C for 30 min, the slides were incubated with HRP‐conjugated streptavidin for 30 min at 37 °C, followed by the incubation with 3,3′‐diaminobenzidine tetra-hydrochloride (DAB) to visualize the positive staining. Then, the slides were counterstained by hematoxylin and the images were captured. Primary antibody was replaced by PBS for negative control.

### Immunofluorescence

The co-expression of TXNIP and MBP in the sciatic nerves of mice was detected by the method of double staining immunofluorescence. In detail, the tissue slides were deparaffinized and hydrated, followed by antigen retrieval using sodium citrate buffer pH 6.0. Nonspecific binding with primary antibodies was prevented by incubating slides with goat serum. The rabbit primary antibody against TXNIP and rat primary antibody against MBP were simultaneously used to incubate slides overnight at 4 °C. Then primary antibodies were detected with goat anti-rabbit labeled DyLight 488 secondary antibody and goat anti-rat labeled DyLight 405 secondary antibody. After rinsed by PBS, slides were observed under laser confocal microscope and images were captured for further analysis. The expression of TXNIP, LC3, cleaved Caspase 3, DNMT1, and DNMT3a in RSC96 cells with the indicated treatment was detected by indirect immunofluorescence. In detail, RSC96 cells seeded on the cover slide in six-well plate were washed three times using PBS, and fixed using 4% paraformaldehyde for 15 min at room temperature. After permeabilized with 0.3% Triton X‐100, cells were incubated with goat serum to prevent non-specific binding. The following antibodies and dilutions were used: TXNIP (1:500), LC3 (1:500), cleaved Caspase 3 (1:200), DNMT1 (1:1000), and DNMT3a (1:200). Then cells were stained with the appropriate fluorophore conjugated secondary antibody and counterstained with 4′,6‐diamidino‐2‐phenylindole (DAPI) to visualize the nuclear and the interesting proteins.

### Western blot

Tissues and cells protein was harvested using RIPA lysis buffer and quantified using a Coomassie brilliant blue protein assay kit. Equal amounts of protein were resolved on a 10% sodium dodecyl sulfate (SDS)‐polyacrylamide gel and electro-transferred to polyvinylidene difluoride (PVDF) membranes. The membranes were incubated with 5% bovine serum albumin (BSA) for 1 h at 37 °C to block non-specific binding. After blocking, the membranes were incubated with specific primary antibodies overnight at 4 °C, followed by the incubation with a secondary antibody conjugated to horseradish peroxidase (HRP) at room temperature for 2 h. The bands were visualized using an enhanced chemiluminescence kit (ECL), after membranes were rinsed by TTBS three times. The β‐actin was used as a control to verify the equal protein loading. The integrated optical density (IOD) ratio of the interesting protein band and β‐actin band was analyzed as the relative expression level.

### Real-time PCR

Real‐time polymerase chain reaction (PCR) was performed according to the instruction. Briefly, total RNA was extracted from the sciatic nerves of mice and in vitro cultured Schwann cells using Trizol. To generate cDNA, equal amounts of total RNA were added to reverse transcription mixture of PrimeScript^TM^ RT reagent Kit with gDNA Eraser. Quantitative real-time PCR was conducted using SYBR® Premix Ex Taq™ II (Tli RNaseH Plus) kit for 40 cycles. Primers for mice TXNIP, mice 18S, rat TXNIP, and rat 18 S were listed in Supplementary Table 1. The expression level of TXNIP mRNA was normalized to 18S mRNA level. The Ct value of each sample was used to calculate the relative expression of TXNIP using the 2^−∆∆Ct^ method. All experiments were independently repeated three times.

### RNA sequencing

Total RNA was isolated from RSC96 cells respectively treated with normal-glucose or high-glucose medium. After purified and fragmented, total RNA was synthesized to first-strand cDNA. Then the second-strand cDNA was produced by PCR. After an adapter was ligated to the end of the cDNA fragment, sequencing was performed on the Illumina HiSeq 2500 (Illumina, San Diego, CA, USA).

### Statistical analysis

All data shown as means ± SD were from at least three independent experiments. Statistical analyses were performed by GraphPad Prism 6 software. For two groups, Student’s *t*-test was used for statistical analyses. For more than two groups, statistical analyses were made using one way Analysis of Variance (ANOVA), followed by the Bonferroni post hoc test. The Kruskal–Wallis test was performed for the comparisons of data with non-normal distribution or heterogeneity of variance. *P* value <0.05 was regarded as a significance.

## Results

### TXNIP expression was increased in the sciatic nerves of diabetic mice

**Fig. 1:**
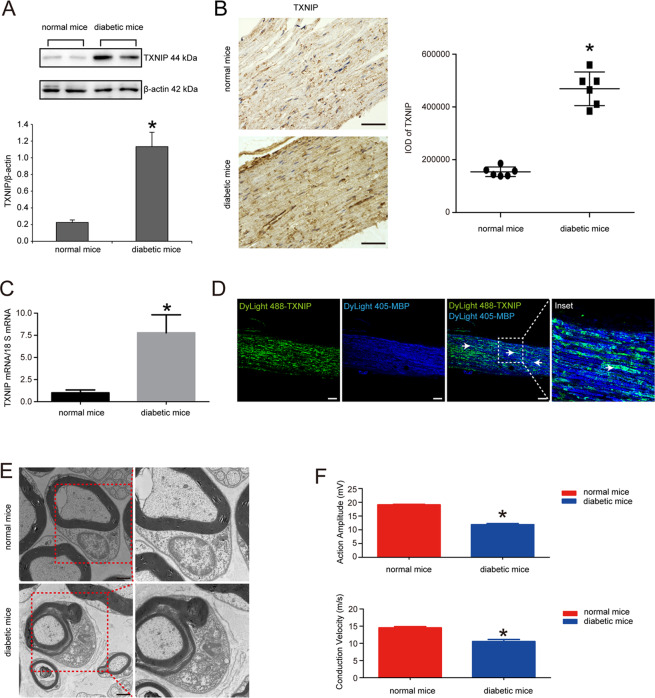
TXNIP expression was increased in the sciatic nerves of diabetic mice. **A** Western blot and statistical analysis of TXNIP expression in the sciatic nerves of normal mice and diabetic mice. *P < 0.05 versus normal mice. **B** Immunohistochemistry of TXNIP expression in the sciatic nerves of normal mice and diabetic mice and the statistical analysis of integrated optical density (IOD) of positive staining. *P < 0.05 versus normal mice. Bar: 50 μm. **C** Real-time PCR of TXNIP mRNA expression in the sciatic nerves of normal mice and diabetic mice. *P < 0.05 versus normal mice. **D** Double staining immunofluorescence of TXNIP (indicated in green) and MBP (indicated in blue) in the sciatic nerves of diabetic mice. White arrows showed the co-localization region. The inset was shown with dashed square. Bar: 50 μm. **E** Electron microscope observation of myelin sheath structure in the sciatic nerves of normal mice and diabetic mice. Bar: 1000 nm. **F** Electrophysiological detection of action amplitude and conduction velocity of the sciatic nerves of normal mice and diabetic mice. *P < 0.05 versus normal mice.

We first investigated the expression of TXNIP in the sciatic nerves of diabetic mice and normal mice by the methods of Western blot, immunohistochemistry and real-time PCR. The results were shown in Fig. 1A that TXNIP protein expression was significantly increased in the sciatic nerves of diabetic mice. Immunohistochemistry results also confirmed that integrated optical density (IOD) of positive region was increased by 67.11% in diabetic mice versus that of normal mice (*P* < 0.05) (Fig. 1B). In line with the above data, real-time PCR also revealed that TXNIP mRNA was increased by 86.88% in the sciatic nerves of diabetic mice *versus* those of normal mice (*P* < 0.05) (Fig. 1C). Double staining immunofluorescence also verified the co-location of TXNIP and myelin basic protein (MBP) known as biomarker of myelinated Schwann cells (Fig. 1D). Again, electron microscopy observation confirmed the abnormality of myelin sheath in diabetic mice, such as infolding and outfolding (Fig. 1E). Furthermore, electrophysiology detection found that action amplitude and conduction velocity were both suppressed in the sciatic nerves of diabetic mice. In detail, action amplitude was decreased by 37.76%, and conduction velocity was decreased by 27.36% (*P* < 0.05) (Fig. 1F).

### High glucose increased TXNIP expression in RSC96 cells, HSC cells, and PRSC cells

**Fig. 2:**
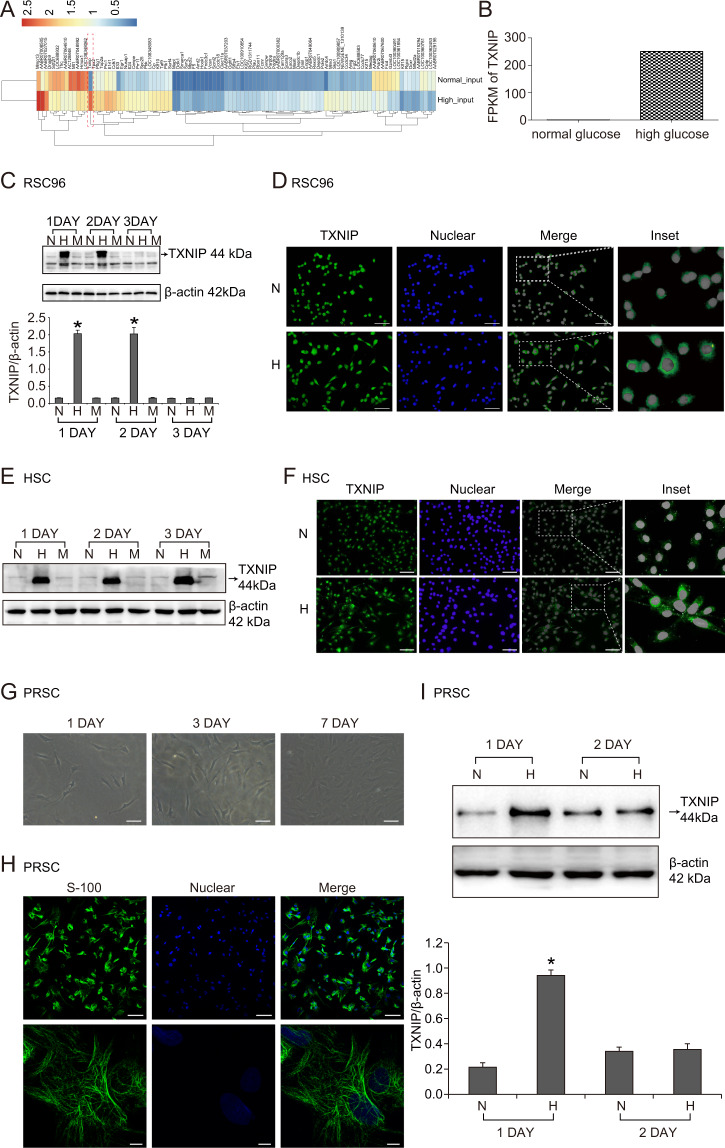
High glucose increased TXNIP expression in RSC96 cells, HSC cells and PRSC cells. **A** RNA-seq results of the main differential expression genes (DEGs) in RSC96 cells treated with normal glucose or high glucose for 48 h. **B** Fragments per kilobase of transcript per million fragments mapped (FPKM) of TXNIP in RSC96 cells stimulated with normal glucose or high glucose. **C** Western blot and statistical analysis of TXNIP expression in RSC96 cells of normal glucose group (5.5 mmol/L glucose, N), high glucose group (25 mmol/L glucose, H) and mannitol group (25 mmol/L mannitol, M) at the indicated time point. *P < 0.05 versus N group. **D** Immunofluorescence of TXNIP in RSC96 cells treated with normal glucose (N) and high glucose (H). The inset was shown with dashed rectangle. Bar: 50 μm. **E** Western blot of TXNIP expression in HSC cells of normal glucose group (5.5 mmol/L glucose, N), high glucose group (25 mmol/L glucose, H) and mannitol group (25 mmol/L mannitol, M). **F** Immunofluorescence of TXNIP in HSC cells treated with normal glucose and high glucose. The inset was shown with dashed rectangle. Bar: 50 μm. **G** The invert microscope observation of primary rat Schwann cells (PRSC) growth. Bar: 50 μm. **H** The identification of PRSC cells by immunofluorescence detection of Schwann cells biomarker S-100. Bar: 50 μm. **I** Western blot and statistical analysis of TXNIP expression in PRSC cells treated with normal glucose and high glucose. *P < 0.05 versus N group.

We further explored the expression of TXNIP in vitro cultured Schwann cell lines treated with high glucose known as the most important diabetic feature. As seen in Supplementary Table 2, the results of RNA-seq showed the top 20 significantly differential expression mRNA between RSC96 cells treated with normal glucose and those treated with high glucose for 48 h. Among those, TXNIP mRNA was markedly increased by 339.87 times (FPKM of high glucose group = 250.38, FPKM of normal glucose group = 0.74) (Fig. 2A, B). Then, western blot detection found that TXNIP protein was respectively significantly increased by 13.47 times and 13.20 times with high glucose treatment for 24 and 48 h in RSC96 cells compared to normal glucose treatment (*P* < 0.05) (Fig. 2C). Similarly, immunofluorescence observation found that TXNIP was located in the nuclear and cytoplasm of RSC96 cells, and high glucose stimulation increased TXNIP expression in the cytoplasm (Fig. 2D). Moreover, we investigated the effect of high glucose on TXNIP expression in human Schwann cells (HSC) and primary rat Schwann cells (PRSC). The results showed in Fig. 2E that TXNIP protein was also enhanced in HSC cells by high glucose treatment for 24, 48, and 72 h. In line, immunofluorescence detection also presented the increased TXNIP expression in the cytoplasm of HSC cells (Fig. 2F). Again, PRSC were cultured from newly born rats (Fig. 2G) and verified by the method of immunofluorescence of S-100 known as Schwann cells marker (Fig. 2H). In addition, TXNIP protein expression was increased by 4.40 times in 24 h high glucose-treated PRSC cells *versus* normal glucose-treated cells (*P* < 0.05) (Fig. 2I).

### TXNIP upregulation mediated high glucose-induced cell autophagy and apoptosis in RSC96 cells

**Fig. 3:**
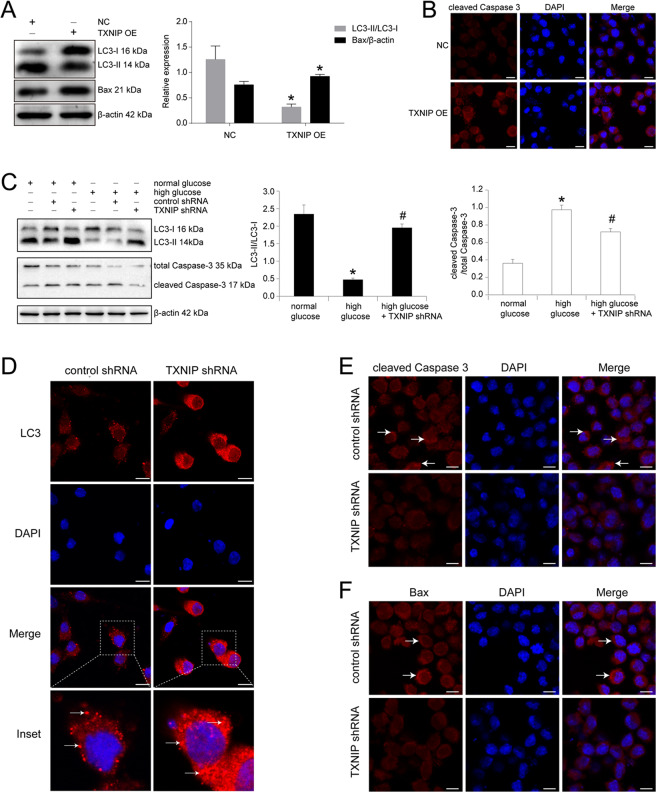
TXNIP upregulation mediated high glucose-induced cell autophagy and apoptosis in RSC96 cells. **A** Western blot and statistical analysis of the effect of TXNIP overexpression (OE) on LC3 and Bax in RSC96 cells. *P < 0.05 versus negative control group (NC). **B** Immunofluorescence of cleaved Caspase 3 in RSC96 cells transfected with negative control plasmid and TXNIP overexpression plasmid. Bar: 10 μm. **C** Western blot and statistical analysis of the effect of TXNIP knockdown using shRNA plasmid (pGenesil-1-TXNIP) on LC3 and cleaved Caspase 3 in RSC96 cells. *P < 0.05 versus normal glucose group. # means *P* < 0.05 versus high glucose group. **D** Immunofluorescence of LC3 in RSC96 cells transfected with control plasmid and TXNIP shRNA plasmid. The inset was shown with dashed square. White arrows showed the clustered LC3 expression. Bar: 10 μm. **E** Immunofluorescence of cleaved Caspase 3 in RSC96 cells transfected with control plasmid and TXNIP shRNA plasmid. White arrows showed the positive expression. Bar: 10 μm. **F** Immunofluorescence of Bax in RSC96 cells transfected with control plasmid and TXNIP shRNA plasmid. White arrows showed the positive signal. Bar: 10 μm.

In addition, we detected the function of TXNIP in high glucose-cultured RSC96 cells. The results were shown in Fig. 3A that upregulation of TXNIP in normal glucose-cultured RSC96 cells decreased LC3-II/LC3-I ratio, as well as increased pro-apoptotic factor Bax expression (*P* < 0.05). The results of immunofluorescence also found that overexpression of TXNIP in RSC96 cells enhanced cleaved Caspase 3 expression indicated in red granules (Fig. 3B). Subsequently, in high glucose-cultured RSC96 cells, knockdown of TXNIP effectively prevented high glucose-induced LC3-II/LC3-I ratio downregulation and cleaved Caspase 3/total Caspase 3 ratio upregulation. Statistical analysis revealed that LC3-II/LC3-I ratio was increased by 4.12 times in TXNIP shRNA plasmid-transfected RSC96 cells versus control shRNA plasmid group (*P* < 0.05). Then cleaved Caspase 3/total Caspase 3 ratio was reduced by 25.94% in TXNIP shRNA plasmid-transfected RSC96 cells versus control shRNA plasmid group (*P* < 0.05) (Fig. 3C). Furthermore, we detected the effect of TXNIP knockdown on autophagy and apoptosis in HSC cells. Similarly, high glucose-induced autophagy inhibition and cell apoptosis were effectively avoided by TXNIP downregulation (Supplementary Fig. 1). In addition, LC3 expression was detected in RSC96 cells by immunofluorescence and it could be seen that compared with control plasmid group the clustered LC3 significantly increased in those cells transfected with TXNIP shRNA plasmid (Fig. 3D). As well, we explored the cleaved Caspase 3 and Bax expression by the method of immunofluorescence, and the results were shown that both cleaved Caspase 3 and Bax were decreased by the downregulation of TXNIP in RSC96 cells (Fig. 3E, F).

### 5-Aza inhibited TXNIP expression in high glucose-treated RSC96 cells and the sciatic nerves of diabetic mice

**Fig. 4:**
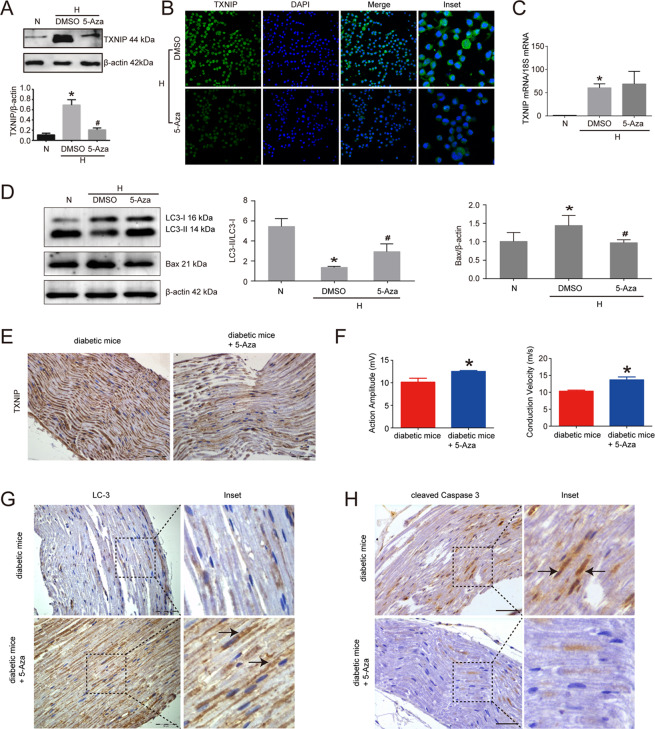
5-Aza inhibited TXNIP expression in high glucose-treated RSC96 cells and the sciatic nerves of diabetic mice. **A** Western blot and statistical analysis of the effect of 5-Aza on TXNIP expression in high glucose-cultured RSC96 cells. *P < 0.05 versus N group. # means *P* < 0.05 versus DMSO group. **B** Immunofluorescence of TXNIP in high glucose-cultured RSC96 cells treated with DMSO and 5-Aza. Bar: 50 μm. **C** Real-time PCR of TXNIP mRNA in high glucose-cultured RSC96 cells treated with DMSO and 5-Aza. *P < 0.05 versus N group. **D** Western blot and statistical analysis of the effect of 5-Aza on LC3 and Bax in RSC96 cells. *P < 0.05 versus N group. #*P* < 0.05 versus DMSO group. **E** Immunohistochemistry of TXNIP in the sciatic nerves of diabetic mice administrated with 5-Aza or not. Bar: 50 μm. **F** Electrophysiological detection of action amplitude and conduction velocity of the sciatic nerves of diabetic mice administrated with 5-Aza or not. *P < 0.05 versus diabetic mice. **G** Immunohistochemistry of LC3 in the sciatic nerves of diabetic mice administrated with 5-Aza or not. The inset was shown with dashed square. Black arrows showed the positive staining of LC3. Bar: 50 μm. **H** Immunohistochemistry of cleaved Caspase 3 in the sciatic nerves of diabetic mice administrated with 5-Aza or not. The inset was shown with dashed square. Black arrows showed the positive staining of cleaved Caspase 3. Bar: 50 μm.

In order to elucidate whether DNA methyltransferase was involved in TXNIP expression in Schwann cells of diabetic peripheral neuropathy, we first explored the effect of 5-Aza known as DNA methyltransferase inhibitor on TXNIP expression in high glucose-cultured RSC96 cells. Then it could be found that 5-Aza treatment markedly suppressed TXNIP protein expression in high glucose-cultured RSC96 cells. Compared with solvent control group, TXNIP expression was decreased by 77.33% in 5-Aza treatment group (*P* < 0.05) (Fig. 4A). In line with the results of Western blot, immunofluorescence also revealed the inhibitory effect of 5-Aza on TXNIP protein in RSC96 cells treated with high glucose (Fig. 4B). Unexpectedly, there was no significant alteration in TXNIP mRNA expression between DMSO-treated group and 5-Aza-treated group in high glucose-cultured RSC96 cells (Fig. 4C). Additionally, 5-Aza treatment also reversed the effect of high glucose on LC3 and Bax (*P* < 0.05) (Fig. 4D).

Furthermore, we explored the in vivo effect of 5-Aza on the sciatic nerves and found that the administration of 5-Aza decreased TXNIP expression in the sciatic nerves of diabetic mice (Fig. 4E), accompanied with the improved conduction velocity and action amplitude of the sciatic nerves (Fig. 4F). Immunohistochemistry results also revealed that LC3 expression was enhanced and cleaved Caspase 3 expression was decreased in the sciatic nerves of diabetic mice treated with 5-Aza (Fig. 4G, H).

### DNMT1 and DNMT3a mediated 5-Aza-inhibited TXNIP expression in high glucose-treated RSC96 cells

**Fig. 5:**
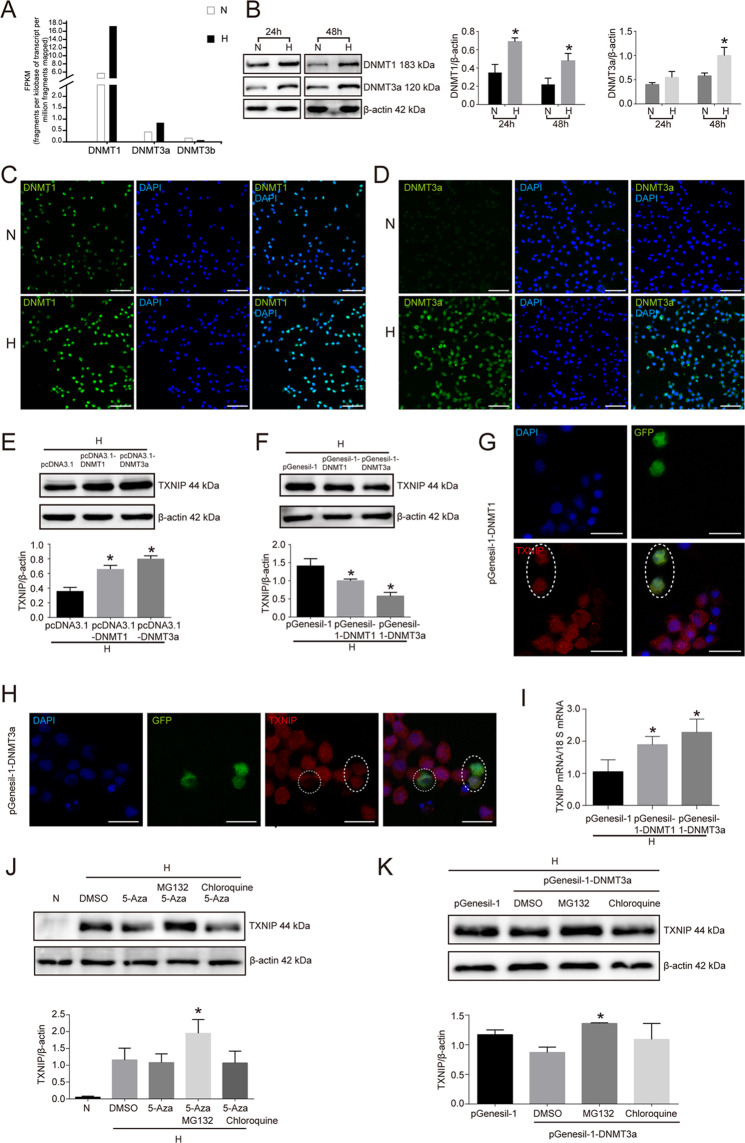
DNMT1 and DNMT3a mediated 5-Aza-inhibited TXNIP expression in high glucose-treated RSC96 cells. **A** RNA-seq of DNMT1, DNMT3a, and DNMT3b in RSC96 cells treated with normal glucose and high glucose. **B** Western blot and statistical analysis of the effect of high glucose on DNMT1 and DNMT3a in RSC96 cells. *P < 0.05 versus N group. **C** Immunofluorescence of DNMT1 in RSC96 cells of normal glucose group (N) and high glucose group (H). Bar: 50 μm. **D** Immunofluorescence of DNMT3a in RSC96 cells of normal glucose group (N) and high glucose group (H). Bar: 50 μm. **E** Western blot and statistical analysis of the effect of DNMT1 and DNMT3a overexpression on TXNIP expression in RSC96 cells. *P < 0.05 versus pcDNA3.1 group. **F** Western blot and statistical analysis of the effect of DNMT1 and DNMT3a knockdown on TXNIP expression in RSC96 cells. *P < 0.05 versus pGenesil-1 group. **G** Confocal microscope observation of TXNIP expression in RSC96 cells successfully transfected with DNMT1 shRNA plasmid (pGenesil-1-DNMT1 expressing GFP tag). Oval showed those cells successfully transfected with pGenesil-1-DNMT1. Bar: 20 μm. **H** Confocal microscope observation of TXNIP expression in RSC96 cells successfully transfected with DNMT3a shRNA plasmid (pGenesil-1-DNMT3a expressing GFP tag). Round and oval showed those cells successfully transfected with pGenesil-1-DNMT3a. Bar: 20 μm. **I** Real-time PCR of TXNIP mRNA in high glucose-cultured RSC96 cells transfected with pGenesil-1, pGenesil-1-DNMT1, and pGenesil-1-DNMT3a. *P < 0.05 versus pGenesil-1 group. **J** Western blot and statistical analysis of the effect of MG132 and chloroquine on TXNIP expression in high glucose plus 5-Aza-treated RSC96 cells. *P < 0.05 versus 5-Aza treatment group. **K** Western blot and statistical analysis of the effect of MG132 and chloroquine on TXNIP expression in DNMT3a knockdown RSC96 cells. *P < 0.05 versus DMSO group.

Considering that DNA methyltransferase is the writer of DNA methylation and the targets of 5-Aza, we further investigated which DNA methyltransferase was involved in 5-Aza-inhibited TXNIP expression in high glucose-cultured RSC96 cells. As illustrated in Fig. 5A, RNA sequencing results showed that DNMT1 mRNA and DNMT3a mRNA were enhanced in RSC96 cells treated with high glucose versus those treated with normal glucose, not DNMT3b. In line, Western blot detection also proved that DNMT1 protein was respectively increased by 49.27 and 54.17% with high glucose stimulation for 24 and 48 h in RSC96 cells. Again, DNMT3a was also respectively enhanced by 26.31 and 42% with high glucose treatment for 24 and 48 h compared to the normal glucose group (Fig. 5B). In addition, immunofluorescence assay also verified the results of Western blot (Fig. 5C, D).

To further demonstrate the direct relationship between DNMT1, DNMT3a and TXNIP in high glucose-cultured RSC96 cells, we transfected RSC96 cells with DNMT1 and DNMT3a expression plasmid (pcDNA3.1-DNMT1 and pcDNA3.1-DNMT3a) and detected TXNIP expression at 48 h after transfection. The results showed that TXNIP protein expression was increased in response to transfection with pcDNA3.1-DNMT1 and pcDNA3.1-DNMT3a compared to pcDNA3.1 in high glucose-cultured RSC96 cells (*P* < 0.05) (Fig. 5E). In turn, high glucose-cultured RSC96 cells transfected with DNMT1 and DNMT3a shRNA plasmid (pGenesil-1-DNMT1 and pGenesil-1-DNMT3a) presented the downregulation of TXNIP protein. (Fig. 5F). Confocal microscope observation also confirmed that RSC96 cells successfully transfected with pGenesil-1-DNMT1 and pGenesil-1-DNMT3a showed the less expression of TXNIP (Fig. 5G, H).

Unexpectedly, knockdown of DNMT1 or DNMT3a in high glucose-cultured RSC96 cells increased TXNIP expression at the level of mRNA. In detail, TXNIP mRNA was increased by 1.8 times in pGenesil-1-DNMT1-transfected cells and 2.16 times in pGenesil-1-DNMT3a-transfected cells versus pGenesil-1-transfected cells (Fig. 5I). These findings suggested that DNMT1 and DNMT3a knockdown decreased TXNIP protein independent of transcription in high glucose-cultured Schwann cells.

Based on the above findings, we speculated that DNMT1 and DNMT3a might regulate TXNIP protein degradation in high glucose-stimulated RSC96 cells. Then, MG132 (proteasome inhibitor) and chloroquine (autophagy inhibitor) were used to treat RSC96 cells-stimulated by high glucose, and the results showed that TXNIP protein suppressed by 5-Aza was effectively reversed by MG132 treatment not chloroquine (Fig. 5J). In addition, DNMT3a knockdown-induced TXNIP expression downregulation was also prevented with MG132 treatment (Fig. 5K).

### PI3K/Akt pathway mediated high glucose-induced DNMT1, DNMT3a, and TXNIP upregulation in RSC96 cells

**Fig. 6:**
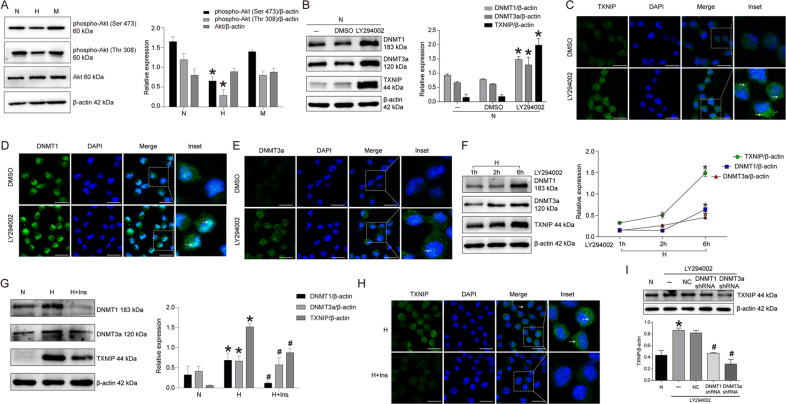
PI3K/Akt pathway mediated high glucose-induced DNMT1, DNMT3a, and TXNIP upregulation in RSC96 cells. **A** Western blot of phospho-Akt (Ser 473), phospho-Akt (Thr 308), and Akt and statistical analysis in RSC96 cells treated with normal glucose, high glucose and mannitol. *P < 0.05 versus N group. **B** Western blot and statistical analysis of DNMT1, DNMT3a, and TXNIP in RSC96 cells treated with LY294002. *P < 0.05 versus DMSO group. **C** Immunofluorescence of TXNIP in RSC96 cells treated with LY294002. The inset was shown with dashed square. White arrows showed positive staining. Bar: 20 μm. **D** Immunofluorescence of DNMT1 in RSC96 cells treated with LY294002. The inset was shown with dashed square. White arrows showed the positive staining. Bar: 20 μm. **E** Immunofluorescence of DNMT3a in RSC96 cells treated with LY294002. The inset was shown with dashed square. White arrows showed the positive staining. Bar: 20 μm. **F** Western blot and statistical analysis of the time-dependent effect of LY294002 on DNMT1, DNMT3a, and TXNIP in high glucose-cultured RSC96 cells. *P < 0.05 versus 1 h group. **G** Western blot and statistical analysis of the effect of insulin on DNMT1, DNMT3a, and TXNIP in RSC96 cells. *P < 0.05 versus N group, # means *P* < 0.05 versus H group. **H** Immunofluorescence of TXNIP expression in high glucose cultured RSC96 cells treated with insulin. The inset was shown with dashed square. White arrows showed the positive staining. Bar: 20 μm. **I** Western blot of the effect of DNMT1 and DNMT3a knockdown on TXNIP expression in LY294002-treated RSC96 cells. *P < 0.05 versus N group, #*P* < 0.05 versus NC group.

PI3K/Akt pathway was an important intracellular signaling pathway that was injured in the Schwann cells of diabetes mellitus. We explored the possible role of PI3K/Akt pathway in high glucose-increased DNMT1, DNMT3a, and TXNIP in Schwann cells. As seen in Fig. 6A that high glucose treatment significantly inhibited Akt phosphorylation at Ser 473 and Thr 308 in RSC96 cells. Again, we inhibited PI3K/Akt pathway in normal glucose-cultured RSC96 cells using LY294002 and found that LY294002 increased DNMT1, DNMT3a, and TXNIP expression (*P* < 0.05) (Fig. 6B). In line with the results of Western blot, immunofluorescence also proved that LY294002 increased DNMT1, DNMT3a, and TXNIP protein expression in RSC96 cells in comparison with DMSO treatment (Fig. 6C–E). Furthermore, the time-dependent experiment of LY294002 revealed that TXNIP showed the same expression tendency to DNMT1 and DNMT3a along with the extension of LY294002 treatment in high glucose-cultured RSC96 cells (Fig. 6F). These findings suggested that PI3K/Akt pathway regulated DNMT1, 3a/TXNIP axis in high glucose-cultured RSC96 cells.

On the contrary, activation of PI3K/Akt pathway using insulin in high glucose-cultured RSC96 cells caused an 82.9% decrease of DNMT1 and a 14.5% decrease of DNMT3a, followed by a 42.33% decrease of TXNIP versus control group (*P* < 0.05) (Fig. 6G). Similarly, immunofluorescence detection also confirmed that insulin treatment evidently reduced TXNIP expression in RSC96 cells treated with high glucose (Fig. 6H). To further elucidate the mediated role of DNMT1 and DNMT3a in PI3K/Akt pathway-regulated TXNIP expression, LY294002-treated RSC96 cells were respectively transfected with DNMT1 shRNA plasmid, DNMT3a shRNA plasmid, and negative control plasmid. The results showed that knockdown of DNMT1 and DNMT3a effectively avoided TXNIP upregulation (Fig. 6I).

### Knockout of TXNIP in vivo ameliorated cell autophagy and improved nerve conduction function in the sciatic nerve of diabetic mice

**Fig. 7:**
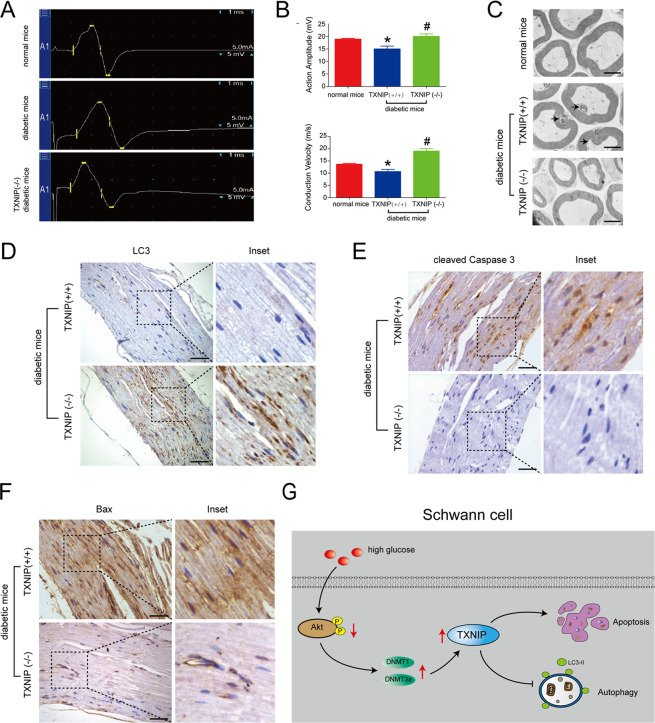
Knockout of TXNIP in vivo ameliorated cell autophagy and improved nerve conduction function in the sciatic nerve of diabetic mice. **A**, **B** Electrophysiological detection of action amplitude and conduction velocity of the sciatic nerves of diabetic mice with *TXNIP* (+/+) and *TXNIP* (−/−). *P < 0.05 versus normal mice. #*P* < 0.05 versus TXNIP (+/+) diabetic mice. **C** Electron microscope observation of myelin sheath in the sciatic nerves of diabetic mice with *TXNIP* (+/+) and *TXNIP* (−/−). Black arrows showed infoldings. Bar: 1000 nm. **D** Immunohistochemistry of LC3 in the sciatic nerves of diabetic mice with *TXNIP* (+/+) and *TXNIP* (−/−). The inset was shown with dashed square. Bar: 50 μm. **E** Immunohistochemistry of cleaved Caspase 3 in the sciatic nerves of diabetic mice with *TXNIP* (+/+) and *TXNIP* (−/−). The inset was shown with dashed square. Bar: 50 μm. **F** Immunohistochemistry of Bax in the sciatic nerves of diabetic mice with *TXNIP* (+/+) and *TXNIP* (−/−). The inset was shown with dashed square. Bar: 50 μm. **G** Model of the regulation of high glucose on the phospho-Akt, DNMTs, TXNIP, apoptosis, and autophagy in Schwann cells.

We further elucidated the in vivo effect of TXNIP on diabetic peripheral neuropathy using TXNIP (−/−) mice receiving STZ for diabetic model construction. It could be illustrated in Fig. 7A, B that compared with diabetic mice (TXNIP +/+), action amplitude was increased by 1.33 times in diabetic mice (TXNIP −/−) (*P* < 0.05). Also, conduction velocity of sciatic nerves was enhanced by 1.78 times in diabetic mice (TXNIP −/−) versus diabetic mice (TXNIP +/+) (*P* < 0.01). Additionally, electron microscope detection showed that the abnormality of myelin sheath of the sciatic nerves in diabetic mice (TXNIP +/+) were prevented by TXNIP knockout (Fig. 7C).

Again, we further detected the expression of autophagy and apoptosis-related proteins in the sciatic nerves of diabetic mice (TXNIP−/−) and diabetic mice (TXNIP+/+). The results of immunohistochemistry showed that the positive LC3 staining in the sciatic nerves of diabetic mice (TXNIP−/−) was more than those of diabetic mice (TXNIP+/+) (Fig. 7D). Similarly, cleaved Caspase 3 and Bax expression was reduced in the sciatic nerves of diabetic mice (TXNIP−/−) versus diabetic mice (TXNIP+/+) (Fig. 7E, F).

## Discussion

In the present study, we first found that TXNIP expression was significantly increased in the Schwann cells of sciatic nerves in diabetic mice, in vitro cultured RSC96 cells, HSC cells and PRSC cells. Similar to our findings, Cheng et al. found that high glucose caused RSC96 cells viability loss, ROS generation, TXNIP expression, and NLRP3 inflammasome activation, followed by IL-1β and IL-18 maturation and gasdermin D cleavage [12]. Also, advanced glycation end products (AGEs) were the detrimental compounds formed by combining protein or fat with sugar, playing an important role in the pathogenesis of diabetic complications [22]. The receptor for advanced glycation end products (RAGE) was reported to induce TXNIP expression in rat primary Schwann cells and the injured sciatic nerve in vivo [23]. Collectively, it was suggested that high glucose and its derivant were the main inducers to elevate TXNIP expression in the Schwann cells of DPN.

In previous studies, autophagy deficiency and apoptosis induction were demonstrated in Schwann cells of DPN [24, 25]. These Schwann cells dysfunctions led to the structural derangement of myelin sheath and the abnormality of peripheral nerves conduction and action amplitude [26, 27]. In this study, we mainly explored the potential effect of TXNIP upregulation on cell autophagy and apoptosis. It was proven that knockdown of TXNIP in RSC96 cells and HSC cells increased LC3-II/LC3-I ratio and decreased cleaved caspase 3/caspase 3 ratio. The relationship between TXNIP and autophagy was also reported in the midbrain of streptozotocin-induced diabetic mice. TXNIP was upregulated by hyperglycemia in the midbrain, accompanied by autophagy inhibition, and dopaminergic (DA) neuron loss. In cultured PC12 cells TXNIP expression was also upregulated by high glucose, and autophagic flux was blocked along with the mitophagy-associated protein PINK1 expression downregulation [28]. In the ischemia/reperfusion myocardial cells, TXNIP expression was reported to inhibit autophagosome clearance [29]. In line with these, TXNIP was also found to regulate cell autophagy in retinal Muller cells of diabetic retinopathy [30] and renal tubular cells of diabetic nephropathy [31]. Also, the relationship between TXNIP and cell apoptosis was previously clarified in various tissues and cells. Song et al. revealed that TXNIP silencing attenuated high glucose-induced apoptosis through obliterating the activation of mTOR and p38 MAPK in mouse podocytes [9]. Similarly, in the hippocampus in female Alzheimer’s disease (AD) mice and amyloid-β 42 (Aβ42)-stimulated SH-SY5Y cells, both apoptosis and TXNIP expression were enhanced. Furthermore, the administration of estradiol significantly inhibited Aβ42-induced cell apoptosis via TXNIP/TRX axis [32]. Therefore, in Schwann cells of DPN TXNIP upregulation affected cell autophagy and apoptosis, and the therapy aimed at TXNIP might be the promising strategy to improve DPN by rescuing Schwann cells dysfunction.

In addition, we investigated the potential mechanism involved in high glucose-enhanced TXNIP expression in Schwann cells. Genome-wide DNA methylation abnormality was clarified to be related with DPN. Guo et al. found that DPN patients with high HbA1c had the evident difference in sural nerve DNA methylome and transcriptome, and total 929 differentially methylated gene (DMGs) were revealed between the highest HbA1c group and the lowest HbAc1 group [33]. In our previous study, we for the first time reported that DNA methyltransferase inhibitor 5-Aza improved BDNF expression in Schwann cells of DPN [17]. Here, we further found that the treatment of 5-Aza decreased TXNIP expression in the sciatic nerves of diabetic mice and in vitro high glucose-cultured RSC96 cells. Also the function of the sciatic nerves of diabetic mice was significantly improved. These data suggested TXNIP expression in the Schwann cells of DPN might be subjected to DNA methyltransferases. Considering DNMT1, DNMT3a and DNMT3b were the downstream targets of 5-Aza, we further analyzed which one was involved in the process of 5-Aza-improved TXNIP expression. The results showed DNMT1 and DNMT3a regulated TXNIP expression, cell apoptosis and autophagy in RSC96 cells. Interestingly, DNMT1 and DNMT3a knockdown reduced TXNIP at the level of protein by regulating protein degradation, not the level of mRNA. TXNIP mRNA upregulation from DNMTs knockdown might derive from the decreased DNA methylation level of TXNIP promoter. Here, we speculated that some potential specific E3 ligases of TXNIP might be involved in DNMTs-regulated TXNIP protein in Schwann cells.

PI3K/Akt pathway plays a pivotal role in cell proliferation, cell cycle control, metabolism, differentiation and apoptosis [34]. Injured PI3K/Akt pathway has been proven in the previous studies on Schwann cell dysfunction in diabetes mellitus [17, 19]. In this study, we revealed the effect of PI3K/Akt pathway on TXNIP in RSC96 cells. The injured Akt phosphorylation was one of the factors to cause TXNIP upregulation in RSC96 cells. The similar regulation of PI3K/Akt pathway on TXNIP was also reported in the study of Song et al. They found inhibition of PI3K/Akt pathway resulted in the TXNIP expression increase in various cell lines including NSCLC cells, leading to a decrease in GLUT1 membrane localization [35]. Similar to our findings that PI3K/Akt inactivation caused an increase in DNMT1 and DNMT3a expression in RSC96 cells, the regulation of PI3K/Akt on DNMT1 and DNMT3a expression was also reported previously. Insulin-like growth factor 1 (IGF1) dramatically increased DNMT1 expression in hepatocellular carcinomas (HCCs) via Akt/GSK-3β signaling pathway activation [36]. GSK-3β phosphorylated DNMT1 at Ser410 and Ser414 to promote DNMT1 protein degradation by increasing the affinity between DNMT1 and the ubiquitin E3 ligase β-transducin repeat-containing protein (βTrCP) [37]. For DNMT3a, the inhibition of GSK3 by PI3K/Akt activation attenuated DNMT3a transcription in mouse embryo stem cells, which led to the hypomethylation of IGF2 and IGF2R [38]. In addition, we also revealed that knockdown of DNMT1 and DNMT3a prevented PI3K/Akt pathway inhibition-upregulated TXNIP in RSC96. Taken together, these findings suggested DNMT1 and DNMT3a mediated high glucose-inhibited PI3K/Akt pathway-caused an increase of TXNIP in Schwann cells of DPN.

In conclusion, this study revealed that TXNIP expression increased in the sciatic nerves of diabetic mice and high glucose-cultured Schwann cell lines. The enhanced TXNIP expression was associated with high glucose-induced autophagy inhibition and apoptosis promotion in RSC96 cells and HSC cells. Furthermore, 5-Aza treatment suppressed TXNIP expression in the sciatic nerves of diabetic mice and high glucose-stimulated RSC96 cells. Moreover, DNMT1 and DNMT3a were demonstrated to regulate TXNIP protein in high glucose-cultured RSC96 cells through affecting protein degradation. Again, the inhibitory PI3K/Akt pathway from high glucose was the reason to lead to DNMT1, DNMT3a and TXNIP upregulation in RSC96 cells (Fig. 7G).

## Availability of data and materials

All data generated in the study are included in this article.

## Supplementary information

Supplementary Figure 1

Supplementary Table 1

Supplementary Table 2
