# Supplementary figures and images for: TXNIP, a novel key factor to cause Schwann cell dysfunction in diabetic peripheral neuropathy, under the regulation of PI3K/Akt pathway inhibition-induced DNMT1 and DNMT3a overexpression
Source: Cell Death Dis. 2021 Jun 23;12(7):642. doi: 10.1038/s41419-021-03930-2 (PMC8222353; doi:10.1038/s41419-021-03930-2)

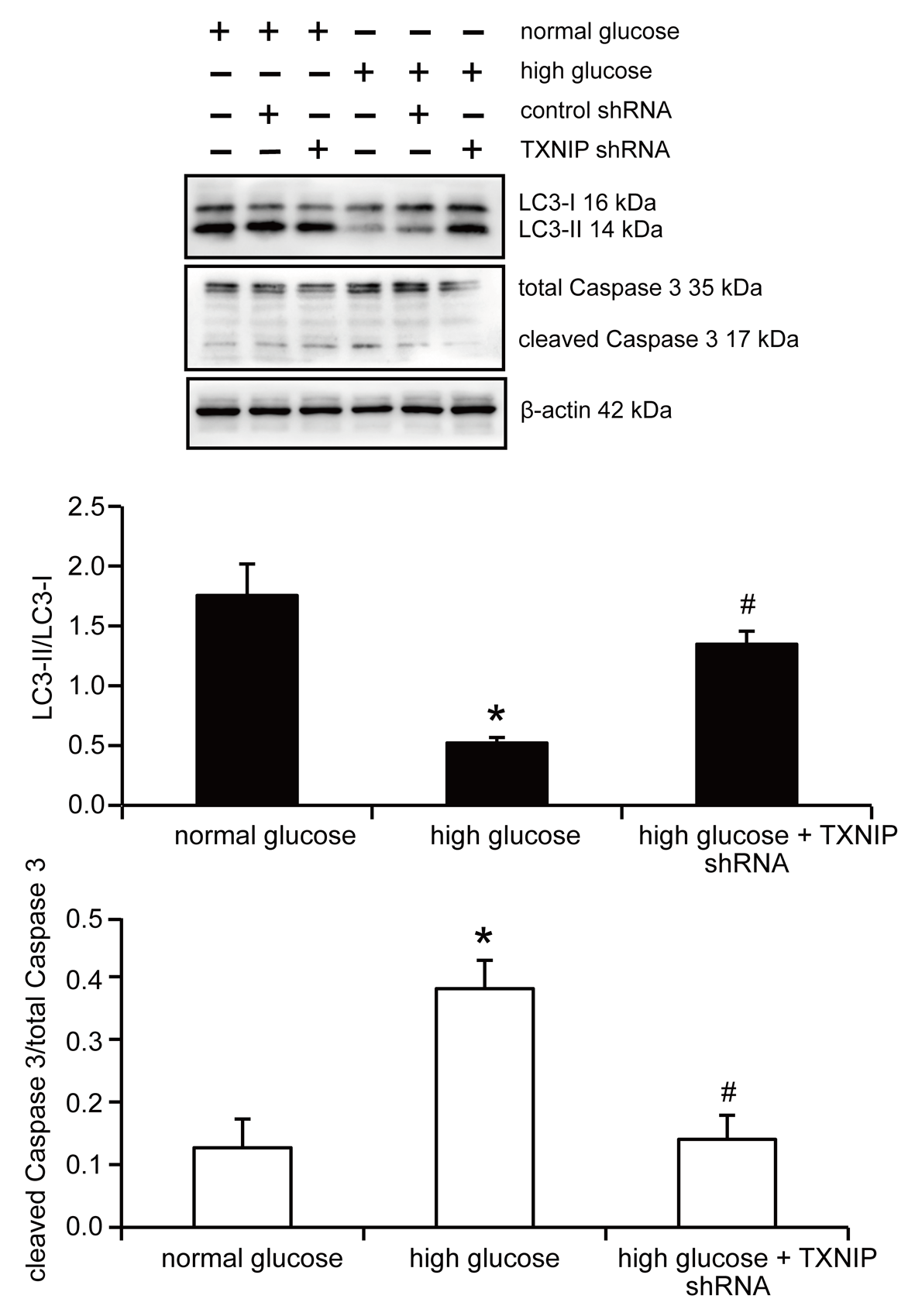

Supplement: Supplementary file 1 — Supplementary Figure 1 [file 41419_2021_3930_MOESM1_ESM.tif]
