# Supplementary material for: TXNIP, a novel key factor to cause Schwann cell dysfunction in diabetic peripheral neuropathy, under the regulation of PI3K/Akt pathway inhibition-induced DNMT1 and DNMT3a overexpression
Source: Cell Death Dis. 2021 Jun 23;12(7):642. doi: 10.1038/s41419-021-03930-2 (PMC8222353; doi:10.1038/s41419-021-03930-2)
Supplement: Supplementary file 2 — Supplementary Table 1 [file 41419_2021_3930_MOESM2_ESM.docx]

**Table.1 PCR primers of TXNIP and 18 S**

|  | **NCBI Reference Sequence** | **Forward Primer** | **Reverse Primer** | **Product** |
| --- | --- | --- | --- | --- |
| Mice TXNIP | NM_001009935.2 | AGTGATTGGCAGCAGGTC | GGTATCTGGGATGTTTAGG | 100 bp |
| Mice 18 S | NM_011296.3 | AAGTTCCAGCACATTTTGCGAGTA | TTGGTGAGGTCGATGTCTGCTTTC | 140 bp |
| Rat TXNIP | NM_001008767.1 | AGTGATTGGCAGCAGGTC | GGTGTCTGGGATGTTTAGG | 100 bp |
| Rat 18 S | NM_213557.1 | AAGTTTCAGCACATCCTGCGAGTA | TTGGTGAGGTCAATGTCTGCTTTC | 140 bp |
