# Supplementary material for: TXNIP, a novel key factor to cause Schwann cell dysfunction in diabetic peripheral neuropathy, under the regulation of PI3K/Akt pathway inhibition-induced DNMT1 and DNMT3a overexpression
Source: Cell Death Dis. 2021 Jun 23;12(7):642. doi: 10.1038/s41419-021-03930-2 (PMC8222353; doi:10.1038/s41419-021-03930-2)
Supplement: Supplementary file 3 — Supplementary Table 2 [file 41419_2021_3930_MOESM3_ESM.docx]

**Table 2 Top 20 differential expression mRNAs in RSC96 cells treated with high glucose**

| **Gene_id** | **Gene_name** | **FPKM (high glucose)** | **FPKM (normal glucose)** |
| --- | --- | --- | --- |
| ENSRNOG00000021201 | **Txnip** | 250.38 | 0.74 |
| ENSRNOG00000010775 | Arrdc4 | 19.38 | 0.36 |
| ENSRNOG00000016456 | Il33 | 48.80 | 1.14 |
| ENSRNOG00000019422 | Egr1 | 43.82 | 2.96 |
| ENSRNOG00000038047 | Mt1 | 16.46 | 215.63 |
| ENSRNOG00000046301 | Gzmbl3 | 4.02 | 42.68 |
| ENSRNOG00000002451 | Fndc3c1 | 2.13 | 0.22 |
| ENSRNOG00000013994 | Enpp1 | 8.71 | 1.00 |
| ENSRNOG00000003762 | Smarca1 | 2.75 | 0.30 |
| ENSRNOG00000001766 | Tfrc | 13.29 | 100.63 |
| ENSRNOG00000001187 | Oasl | 9.21 | 1.15 |
| ENSRNOG00000027002 | NEWGENE_1310139 | 11.18 | 1.46 |
| ENSRNOG00000002436 | Mmd | 16.71 | 2.18 |
| ENSRNOG00000005902 | Ccdc34 | 13.84 | 1.85 |
| ENSRNOG00000029212 | Vcan | 1.93 | 0.27 |
| ENSRNOG00000004441 | Fcmr | 5.27 | 0.72 |
| ENSRNOG00000002403 | Fam129a | 6.74 | 0.99 |
| ENSRNOG00000057855 | F5 | 1.33 | 0.19 |
| ENSRNOG00000016177 | Scara3 | 6.74 | 1.01 |
| ENSRNOG00000006198 | Prr11 | 21.78 | 3.31 |
